# PROFAST: A Randomized Trial Assessing the Effects of Intermittent Fasting and *Lacticaseibacillus rhamnosus* Probiotic among People with Prediabetes

**DOI:** 10.3390/nu12113530

**Published:** 2020-11-17

**Authors:** Audrey Tay, Hannah Pringle, Elise Penning, Lindsay D. Plank, Rinki Murphy

**Affiliations:** 1 Department of Medicine, University of Auckland, Auckland 1142, New Zealand; audrey.tay@auckland.ac.nz; 2 Discipline of Nutrition & Dietetics, Faculty of Medical and Health Sciences, University of Auckland, Auckland 1142, New Zealand; hpri988@aucklanduni.ac.nz (H.P.); epen392@aucklanduni.ac.nz (E.P.); 3 Department of Surgery, University of Auckland, Auckland 1142, New Zealand; l.plank@auckland.ac.nz

**Keywords:** intermittent fasting, probiotics, *Lacticaseibacillus rhamnosus* HN001, prediabetes

## Abstract

Both intermittent fasting and specific probiotics have shown promise in improving glucose tolerance with a potential for synergistic effects through alterations to gut microbiota. In this randomized, double-blinded, two-arm feasibility study, we investigated whether intermittent fasting, supplemented with *Lacticaseibacillus rhamnosus* HN001 probiotic, reduces HbA1c in individuals with prediabetes. All participants with HbA1c 40–50 mmol/mol commenced intermittent fasting (2 days per week of calorie restriction to 600–650 kcal/day) and were randomized 1:1 to either daily probiotic (*Lacticaseibacillus rhamnosus* HN001) or placebo for 12 weeks. The primary outcome was a change in HbA1c. Secondary outcomes included changes in anthropometry, body composition, glucoregulatory markers, lipids, hunger hormones, liver enzymes, inflammatory markers, gut hormones, calorie and macronutrient intake, quality of life, hunger, mood and eating behavior. Of 33 participants who commenced the trial, 26 participants (mean age 52 years, body mass index (BMI) 34.7 kg/m^2^) completed the intervention (*n* = 11 placebo, *n* = 15 probiotic). HbA1c decreased from 43 ± 2.7 mmol/mol to 41 ± 2.3 mmol/mol, *p* < 0.001, with average of 5% weight loss. No significant between-group differences were seen in primary or secondary outcomes except for social functioning (*p* = 0.050) and mental health (*p* = 0.007) scores as improvements were seen in the probiotic group, but not in the placebo group. This study shows additional psychological benefits of probiotic supplementation during intermittent fasting to achieve weight loss and glycemic improvement in prediabetes.

## 1. Introduction

The global prevalence of diabetes is set to increase within the next 10 years, affecting 439 million adults by 2030 [1]. The overall prevalence of diabetes in New Zealand is 7%, with an additional 17% with prediabetes [2]. Individuals with prediabetes are at an increased risk of cardiovascular disease [3], and 5–10% of those with prediabetes transition to develop type 2 diabetes each year [4]. Therefore, prevention interventions that are cost-effective and acceptable are crucial.

Recent research into very-low-calorie diets [VLCD; 400–800 kcal/day] for prevention or reversal of type 2 diabetes has shown success [5,6,7]. Although VLCD is successful in achieving weight loss and subsequent improvement in glycemic control, this is largely dependent on the participants’ ability to adhere to dietary guidelines once the intensive meal-replacement phase is transitioned to the maintenance diet [8]. A promising alternative to continuous energy restriction is intermittent fasting, which has been found to achieve weight loss goals with improved adherence [9]. Intermittent fasting is an umbrella term for various diets that cycle between a period of fasting and non-fasting over a defined period [10]. The intensity of fasting can vary from total absence of calories in a water-only fasting day to a reduction of calories of varying extent. Studies have found a significant reduction in glucoregulatory markers after implementing intermittent fasting [11,12]. Furthermore, some intermittent fasting trials reported no change in self-reported hunger levels [12,13]. In contrast, VLCD interventions have found an increase in ghrelin levels, an appetite-stimulating hormone [14]. As hunger is a common precursor for weight regain, this suggests that intermittent fasting may achieve more sustained weight loss. The 5:2 diet consists of two days of reduced caloric intake to 600–650 kcal/day per week and five days of ad libitum eating. This form of intermittent fasting may suit individuals who struggle with continuous daily modest calorie restriction (i.e., 1200 kcal/day) or VLCD (400–800 kcal/day) and may therefore be a feasible weight loss approach with accompanying improvements in weight-related complications.

There is growing evidence linking gut dysbiosis with obesity and diabetes. Animal studies have suggested that probiotics can improve insulin sensitivity and beta-cell dysfunction by regulating the key signaling pathways [15,16]. Specific strains from the *Lactobacillus* and *Bifidobacterium* genera have been named the most effective for glucose and weight control [17]. Recent evidence from a New Zealand study showed that the probiotic *Lacticaseibacillus rhamnosus* HN001 (previously known as *Lactobacillus rhamnosus* HN001) can result in improved gestational diabetes prevalence despite no changes in body weight [18]. Other research has linked probiotics to improvements in inflammation [19] and other biomarkers of oxidative stress [20]. Multiple studies have investigated the synergistic effect of a probiotic intervention with a hypocaloric diet and have reported augmented improvements in health markers [21,22,23]. However, no studies have examined the effect of probiotics in combination with intermittent fasting.

Both intermittent fasting and probiotic supplementation show favorable health outcomes in their own right; however, combining the two may augment these benefits. The PRObiotics and intermittent FASTing to improve prediabetes (PROFAST) trial aimed to establish whether intermittent fasting and daily probiotic supplementation can improve HbA1c in individuals with prediabetes. To our knowledge, this is the first study to combine these two intervention methods. The results of this study will contribute to the growing body of evidence regarding intermittent fasting and probiotic supplementation for the prevention of type 2 diabetes.

## 2. Materials and Methods

### 2.1. Trial Design

The PROFAST trial was a 12-week, double-blinded, two-armed, randomized 1:1 study of daily probiotic vs. placebo in the presence of intermittent fasting. This study was a pilot, feasibility study; hence no power calculation was done. This study is registered on the Australian New Zealand Clinical Trials Registry, registration number: ACTRN12616001050448.

### 2.2. Participants

All participants provided informed consent for inclusion before they participated in the study. The study was conducted in accordance with the Declaration of Helsinki, and the protocol was approved by the Health and Disability Ethics Committee (16/STH/107) on 12 August 2016.

Participants were either referred to the PROFAST trial by primary care clinicians or self-selected to participate. Eligible participants were aged between 18 and 65 years, had prediabetes (defined as glycated hemoglobin (HbA1c) of 40–50 mmol/mol in the preceding 9 months), and a body mass index (BMI) of 30–40 kg/m^2^ (or 27–40 kg/m^2^ for Indian ethnicity). Participants were excluded if they had pre-existing diabetes, previous bariatric surgery, were currently taking glucose-lowering medications, had any conditions that may influence body weight regulation (e.g., thyroid disorders, malabsorption, eating disorders, use of systemic steroids, excess alcohol intake), or had any significant health issues (e.g., end-stage renal failure, congestive heart failure, thalassemia, psychiatric disease). Women were excluded if they were pregnant, breastfeeding, or intending to become pregnant. Participants were also excluded if they were planning any major changes in physical activity during the study to the extent that may interfere with the study outcomes, donated blood within 2 months before the study or experienced a weight change greater than 3 kg within 3 months of the first baseline visit.

### 2.3. Interventions

The probiotic and placebo capsules were provided by Fonterra Cooperative Group, Ltd. (Auckland, New Zealand), who had no role in the design of the study; in the collection, analyses or interpretation of data; in the writing of the manuscript; or in the decision to publish the results. Capsules had identical packaging and were labeled A or B. The probiotic capsule contained the strain *L. rhamnosus* HN001 at a dose of 6 × 10^9^ colony forming units (CFU). The placebo capsule contained microcrystalline cellulose and dextrose anhydrate. Participants were given 90 capsules (12 weeks supply) of either the probiotic or placebo.

The intermittent fasting regimen consisted of eating ad libitum for five days and then restricting calories (‘fasting’) for two days per week, for a duration of 12 weeks. On the fasting days, participants were instructed to restrict their calories to a maximum of 600 kcal/day for females and 650 kcal/day for males. Before commencing the 3month intervention, participants were provided with dietary advice and education regarding this fasting regime by the study dietitian. This included education around label reading, calorie counting and how to manipulate recipes to increase satiety while restricting calories. Behavior change techniques were also utilized, such as problem-solving, goal setting and self-monitoring. Participants were encouraged to increase their intake of vegetables and protein on the fasting days and to eat like “normal” on the remaining five days. Physical activity was advised to be kept to the same duration, intensity and frequency as before the study; however, compliance was not measured. Participants received personalized advice and encouragement from the study dietitian on a fortnightly basis, either via phone call, text message or email. To provide additional support, participants were encouraged to contact the study dietitian when required and join a private Facebook group for online discussions.

### 2.4. Randomization and Blinding

Following the baseline visit, eligible participants were randomized to either intermittent fasting with daily probiotic or intermittent fasting with daily placebo in a 1:1 ratio. This was achieved using opaque sealed envelopes, given by the research assistant, which were labeled either treatment group A or B. The randomization process was determined by a computer-generated variable block size random enumeration, stratified for ethnicity (European, Indian, Māori, Pacific) and BMI (<35, ≥35 kg/m^2^).

Study participants and the research team were blinded to the probiotic/placebo allocation during the study. Those who analyzed the data were blinded to group assignments. This information was only released to the investigators when all data had been collected and finalized. Both placebo and probiotic-containing capsules were identical in appearance and packaging.

### 2.5. Outcomes

The primary outcome was change in HbA1c after 12 weeks. Secondary outcomes included changes in: anthropometric and body composition measurements, resting energy expenditure (REE), calorie and macronutrient intake, lipid measurements, liver function tests, gut hormones (ghrelin, GIP, active GLP-1, glucagon and PYY), inflammatory markers, glucoregulatory markers (glucose, insulin, C-peptide), mood and eating questionnaire scores, self-reported hunger and satiety and quality of life questionnaire (SF-12) scores.

### 2.6. Data Collection

At baseline (week 0) and conclusion (week 12), participants attended the Body Composition Laboratory (University of Auckland, New Zealand) after an overnight fast for assessment. This included blood collection for the primary outcome of HbA1c and a 6-point oral glucose tolerance test (OGTT), anthropometry, body composition measurements using dual-energy X-ray absorptiometry (model iDXA, GE-Lunar, Madison, WI) and resting energy expenditure by indirect calorimetry (TrueOne 2400, Parvo Medics, Salt Lake City, UT). Participants completed a visual analog scale (VAS) questionnaire to determine their perceived levels of hunger and fullness before ingesting the 75 g glucose drink for the OGTT. Four questions were included in the VAS questionnaire: “How hungry are you?”, “How full do you feel?”, How strong is your desire to eat?”, “How much food could you eat?” [24]. The SF-12 questionnaire was used to assess quality of life and was completed by each participant at baseline and 12 weeks follow-up. The SF-12 consisted of 12 questions covering 8 health domains related to physical and mental health (physical function, role physical, bodily pain, general health, vitality, social functioning, role emotional and mental health) [25].

All participants completed a mood and eating questionnaire adapted from three validated questionnaires: PHQ-9 [26], generalized anxiety disorder (GAD) questionnaire [27] and the Eating Disorders Questionnaire [28]. Participants were required to bring a completed 7-day food diary to the baseline visit to ascertain their baseline calorie and macronutrient intake. Participants were instructed to complete a food diary for each of their fasting days throughout the 3-month intervention and to present a 7-day food diary at each clinic visit. The use of smartphone applications, such as MyFitness Pal and Easy Diet Diary, was encouraged to more accurately calculate calorie intake.

A subsample of participants (*n* = 14) was invited to complete a post-intervention interview with the study dietitian. Data were recorded on a standardized questionnaire and included topics such as dietary habits before, during and after the intervention; any changes in physical activity, adherence to the study protocol, food choices on fasting days, antibiotic use and experienced side effects. Qualitative data were also obtained on the acceptability of the intervention and whether participants would continue with the intermittent fasting regime post-intervention.

### 2.7. Statistical Analysis

Statistical analyses were performed using SAS version 9.4 (SAS Institute, Cary, NC, USA). All statistical tests were two-sided at a 5% significance level. Repeated measures analysis of variance (treatment group as a fixed factor, time as a repeated factor) was used to assess the effect of treatment for normally distributed data. When the group x time interaction was significant, post hoc analysis using paired t-tests was conducted to establish the significance of within-group changes. For non-normally-distributed data, the Mann–Whitney U test was used to compare changes between the groups and the Wilcoxon signed-ranks test for within cohort changes. Food diaries were analyzed using Foodworks^®^ 7 (Xyris^TM^ Software), which used nutritional data from FOODfiles [29].

## 3. Results

### 3.1. Recruitment

**Figure 1:**
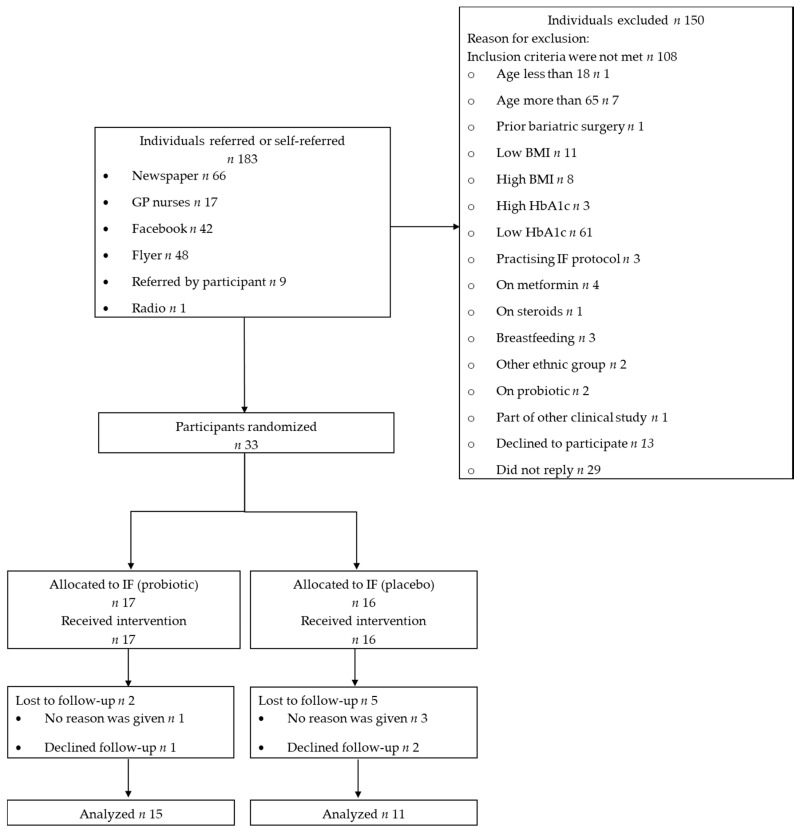
Consort diagram detailing participant numbers from recruitment to analysis. Abbreviations: BMI, body mass index; IF, intermittent fasting; GP, General Practice.

This study was conducted between December 2016 and December 2017, as determined by our feasibility grant. Figure 1 outlines the process from participant screening to analysis. Of the 33 participants randomized into this trial (17 in the probiotic group and 16 in the placebo group), seven participants were lost to follow-up (two from the probiotic group and five from the placebo group). Therefore, the final analysis included 26 participants, with 15 in the probiotic group and 11 in the placebo group.

### 3.2. Baseline Data

All participants had prediabetes, with a mean HbA1c of 43 mmol/mol (range: 40–48 mmol/mol) and a mean BMI of 35 kg/m^2^ (range: 29–42 mmol/mol). The baseline characteristics of all participants randomized into this trial are summarized in Table 1. There were no significant differences between the two groups for baseline data.

### 3.3. Outcomes

A summary of post-intervention results for primary and secondary outcomes are presented in Table 2.

#### 3.3.1. Glucose and Biochemistry

After 12 weeks of intermittent fasting, mean HbA1c for the combined groups (*n* = 26) reduced by 2 mmol/mol from 43.0 ± 2.7 mmol/mol to 41.0 ± 2.3 mmol/mol (*p* < 0.001). There was no difference between the probiotic or placebo groups (Table 2), nor between males and females. HbA1c decreased in 23 out of the 26 participants (88%) and 3 participants (12%) achieved normoglycemia (HbA1c < 40 mmol/mol).

Fasting glucose, insulin and C-peptide levels remained unchanged after the intervention (Table 2). During the OGTT, there was a significant decrease in the area under the curve between baseline and 120 min (AUC_0-120 min_) for glucose when both groups were combined (*p* = 0.020). However, there was no statistically significant difference observed between the probiotic and placebo groups (*p* = 0.056). Insulin levels in the combined group decreased after 12 weeks of intermittent fasting at 90 min, which was the peak insulin timepoint during the OGTT. There was no treatment effect on any lipid measurements (cholesterol, triglycerides, high density lipoprotein (HDL), low density lipoprotein (LDL)). Leptin levels decreased after the 3-month study period (*p* = 0.045), with no differences with probiotic supplementation. No significant differences were observed for liver enzymes (aspartate transaminase (AST) and alanine aminotransferase (ALT)), nor inflammatory markers (interleukin-6 (IL-6) and tumor necrosis factor- α (TNF-α)).

#### 3.3.2. Weight and Body Composition

Both groups lost a similar amount of weight (*p* < 0.001, Table 2). There was no difference in weight loss between men and women. Across all completers, waist circumference and waist-to-hip ratio decreased (*p* < 0.001 and *p* = 0.025, respectively). There were no significant differences between the two groups for these measures. There was also a significant reduction in total fat mass (*p* < 0.001), abdominal fat (*p* < 0.001), visceral fat (*p* < 0.001) and fat-free mass (*p* = 0.005) across all completers, with no difference observed between groups.

#### 3.3.3. Resting Energy Expenditure

REE decreased post-intervention (*p* = 0.007), with no difference between groups (Table 2). Using a prediction equation generated from baseline data, in which the only significant predictor was fat-free mass (FFM), we predicted an REE at 12 weeks equal to 1647.9 (SEM 61.2) kcal/d. This was significantly higher than the measured REE at 12 weeks by 71.5 (SEM 31.8) kcal/d (*p* = 0.034).

#### 3.3.4. Dietary Intake

Energy intake was estimated from completed food diaries and is shown in Table 3. At baseline, participants’ average daily caloric intake was 2310 ± 603 kcal. At the end of the 3-month intervention, average caloric intake was reduced to 1743 ± 507 kcal per day, including fasting days (*p* = 0.002). The average caloric intake on fasting days was 755.4 ± 207.8 kcal in the placebo group and 651.6 ± 147.7 kcal in the probiotic group, with no difference between groups (*p* = 0.206). Women reported intake, which exceeded the set calorie allowance on fasting days (average intake of 711.5 kcal, the protocol was 620 kcal), whereas men reported intake was below recommended (average intake of 620 kcal, the protocol was 650 kcal). As expected, the intake of all macronutrients (fat, carbohydrate and protein) also reduced significantly; however, the percentage of daily intake from each of these macronutrients remained similar. When fasting days were excluded from the analysis, a tendency to reduce was seen in average daily caloric intake (*p* = 0.058). The frequency of self-reported overeating and number of days when overeating occurred were also reduced after the intervention (*p* = 0.007, *p* = 0.032, respectively). There were no differences between the probiotic and placebo groups for either of these measures.

#### 3.3.5. Psychological Outcomes

Psychological outcomes at baseline were similar between groups (*p* = 0.424). When all participants were combined, depression scores improved (*p* = 0.003); however, there was no statistically significant difference between the probiotic and placebo groups (*p* = 0.845). There was an improvement in mental health scores in the probiotic group (*p* = 0.001), and no improvement was seen in the placebo group. This resulted in a statistically significant difference between the two groups (*p* = 0.007). Similarly, scores for social functioning also improved in the probiotic group (*p* = 0.007) with no change in the placebo group with the difference between groups reaching the 5% level of significance. Participants reported an overall improvement in general health after the intervention (*p* = 0.013), but no difference between the groups was observed. There were no differences between groups or between baseline and 12 weeks post-intervention observed for the other five domains (physical functioning, role physical, bodily pain, vitality and role emotional) (Table 4).

#### 3.3.6. Side Effects

Eleven participants (48%) reported mild side effects such as headaches (17%), dizziness/nausea (13%), feeling irritable due to hunger or "hangry" (13%), reduced concentration (4%), increased hunger (4%), feeling grumpy (4%) or general malaise (4%). These did not differ by probiotic versus placebo allocation.

#### 3.3.7. Ancillary Analyses

Participants preferred to be contacted by text message or email during the 3-month intervention. There was no correlation between the frequency of contact and HbA1c reduction or weight loss. A subsample of participants (*n* = 14) provided qualitative data on their experiences during this intervention. All participants recognized that psychological readiness was a major factor in their ability to adhere to the diet, while motivation and commitment were the keys to success. Participants were asked to rate how different their baseline diet compared with their diet at 12 weeks on a scale from one to ten (one = no change, ten = huge change) and scored the extent of diet change as 5.2 ± 2.8. Similarly, participants rated the ease of adhering to the diet on a scale from one to ten (1 = very easy, 10 = very difficult) and scored the experience 3.8 ± 2.0. Five participants (36%) found it difficult to navigate social events due to the food environment, social pressures, temptations, and the added challenge of calorie counting preprepared mixed foods. Twelve participants (86%) reported an increased awareness of their eating behaviors and foods consumed after this intervention, and 13 participants (93%) reported that they would continue with this intermittent fasting regime after the study’s completion.

## 4. Discussion

The PROFAST trial showed that daily supplementation of *Lacticaseibacillus rhamnosus* HN001 probiotic in individuals with prediabetes who practiced intermittent fasting did not result in additional glycemic improvement or weight loss when compared with placebo. However, those supplemented with HN001 showed greater improvements in mental health scores compared to those supplemented with placebo, and this between-group difference was statistically significant. The lack of significant differences between groups for weight loss and change in HbA1c suggests that the greater mental health improvements in the probiotic group were not attributed to these factors. Our results, therefore, offer new evidence that probiotics paired with intermittent fasting can improve mental health. Another New Zealand study investigated the effect of the same probiotic *L. rhamnosus* HN001 versus placebo on postnatal mood in women with gestational diabetes and found that daily probiotic supplementation was associated with lower depression and anxiety scores in the postpartum period [30]. Animal studies have also consistently shown that probiotics positively impact anxiety-and depressive-like behaviors [31]. Although obesity is associated with depressive and anxiety disorders [32], we did not compare our participants’ scores with those of a normal New Zealand population. Further research is needed to explore the effects of probiotic supplementation and intermittent fasting among participants with depressive and anxiety disorders.

This trial also adds to emerging evidence that intermittent fasting can reduce body weight and HbA1c. Weight loss in individuals with prediabetes, whether achieved via dietary or surgical methods, is consistently accompanied by reductions in HbA1c [33]. The magnitude of HbA1c reduction is often proportional to the baseline HbA1c, as greater improvements are achievable from higher HbA1c. In our study, eligible participants had an HbA1c of 40–50 mmol/mol (prediabetes), so the observed 2 mmol/mol reduction may appear modest when compared to other trials that recruited individuals who had type 2 diabetes and thus a higher baseline HbA1c [12,34]. Nevertheless, in view of other diabetes prevention trials, such a reduction in HbA1c represents considerable clinical success [35] and is comparable to results of commercial weight management programs among people with prediabetes [36]. A systematic review and meta-analysis of 36 studies assessing type 2 diabetes prevention in real-world settings suggest that modest reductions in weight (1.6 kg) and HbA1c (2 mmol/mol) can significantly reduce progression to type 2 diabetes compared to usual care by 26% [36].

The 5% weight loss achieved through intermittent fasting is close to the recommendation of 7% weight loss for diabetes prevention by the American Diabetes Association [37]. This was achieved in 12 weeks and required little time investment from the study team as participants were followed-up via text message, phone call or email on a fortnightly basis. There was no additional weight loss or HbA1c reduction with probiotics, which highlights that clinically significant weight loss can occur with two main components–dietary manipulation and regular virtual support. Unfortunately, there were insufficient resources to continue monitoring participants beyond the 12-week intervention, and thus we do not know whether weight loss and improvements in HbA1c were maintained once regular support was removed. We also cannot conclude that these results were due solely to intermittent fasting, as there was no comparison group using continuous energy restriction. Further research is also needed to establish whether this short-term intermittent fasting intervention can produce sustained weight loss and glycemic improvements.

As with all interventions, outcomes are dependent on participants’ adherence to nutritional guidelines. Participants reported a moderate change between their baseline diet and their diet post-intervention. Participants also reported an overall increased awareness of eating behavior. Although pretreatment binge eating disorder was not assessed, participants reported fewer episodes of overeating during the intervention. This hints that adopting an intermittent fasting regime may have more positive than negative impacts on eating behavior. A systematic review published in 2015 found inconsistent associations between severe dietary energy restriction (i.e., low or very low energy diets) and the onset of binge eating in individuals without pretreatment binge eating [38]. This, therefore, affirms that calorie restriction may not necessarily trigger adverse eating behaviors in those without binge eating disorder; however, pretreatment screening is still advised.

The main limitations of this study are the small sample size, the lack of an intention-to-treat analysis and the absence of a comparison group using continuous energy restriction. Consequently, we are unable to recommend intermittent fasting over the traditional method of continuous energy restriction in clinical settings. Nevertheless, our results suggest that intermittent fasting may result in weight loss and diabetes prevention, which should be studied further. The results of the secondary analyses need to be interpreted cautiously owing to the multiplicity of tests conducted. Controlling for false discovery rate using Benjamini–Hochberg procedures [39] yields the smallest p value of 0.392 (instead of 0.007 for mental health).

Several factors were not controlled for in this study, which may have influenced results. First, participants were instructed to maintain their habitual exercise patterns throughout the intervention period; however, this was not monitored to ensure compliance. If participants did alter their exercise levels, our observed results might not be due to intermittent fasting alone. Future studies could perhaps include specific exercise guidance and monitoring, which may also limit the reduction in fat-free mass and resting energy expenditure observed in the present study.

Given the emphasis on restricting calories ascribed to this intervention, the validity and accuracy of using self-reported food diaries to measure caloric intake are limited. Participants may have underreported caloric intake according to social desirability bias [40] or reactively changed their eating behavior to appear more adherent to the PROFAST protocol. Emerging evidence also suggests that dietary fiber can improve the functionality of probiotics, owing to a synbiotic approach [41]. Although participants were encouraged to increase their fiber intake to increase satiety on fasting days, this was not adequately controlled for in our study and may have influenced our results.

## 5. Conclusions

The PROFAST randomized controlled trial demonstrates that *Lacticaseibacillus rhamnosus* probiotic does not have any additional benefits to weight loss or diabetes prevention when combined with an intermittent fasting intervention. Our results suggest that intermittent fasting may result in weight loss and diabetes prevention, which should be studied further. The key finding of this study is that the probiotic *L. rhamnosus* further improves social functioning and mental health outcomes when used together with intermittent fasting among people with prediabetes. This, therefore, presents an opportunity for future research to further explore the psychological benefits of probiotics in this context.

## Figures and Tables

**Table 1:** Baseline characteristics for participants according to intervention allocation and completion status.

Variable	IF (Placebo)	IF (Probiotic)	All Completers	Non-Completers
No. of participants	11	15	26	7
Age (years)	54.1 ± 6.4	52.9 ± 8.7	53.4 ± 7.6	48 ± 8.8
Gender				
Male *n* (%)	2 (18)	6 (40)	8 (31)	1 (14)
Female *n* (%)	9 (82)	9 (60)	18 (69)	6 (86)
Ethnicity				
NZ European *n* (%)	6 (55)	6 (40)	12 (46)	2 (29)
Māori *n* (%)	1 (9)	3 (20)	4 (15)	3 (43)
Pacific *n* (%)	1 (9)	0 (0)	1 (4)	1 (14)
Indian *n* (%)	3 (27)	6 (40)	9 (35)	1 (14)
HbA1c (mmol/mol)	42.9 ± 2.6	43.1 ± 2.9	43.0 ± 2.7	45.4 ± 3.3
Anthropometry				
Weight (kg)	91.3 ± 13.9	98.6 ± 18.4	95.5 ± 16.8	100.5 ± 15.3
Height (cm)	164.7 ± 7.4	168.4 ± 11.7	166.8 ± 10.1	165.1 ± 11.1
BMI (kg/m^2^)	33.6 ± 3.7	34.7 ± 4.9	34.2 ± 4.4	36.7 ± 2.4
Waist (cm)	111.1 ± 11.0	112.7 ± 16.3	112.0 ± 14.1	112.5 ± 10.0
Hip (cm)	116.3 ± 9.8	122 ± 13.1	119.6 ± 12.0	122.5 ± 7.5
WHR	0.96 ± 0.1	0.92 ± 0.1	0.94 ± 0.1	0.90 ± 0.10
Neck (cm)	39.1 ± 4.2	40.9 ± 3.8	40.1 ± 4.0	41.0 ± 4.0

Data are presented as mean ± standard deviation or number of participants (percentage). Abbreviations: BMI, body mass index; IF, intermittent fasting; NZ, New Zealand; HbA1c, glycated hemoglobin; WHR, waist-to-hip ratio.

**Table 2:** Comparison of baseline and post-intervention (12 weeks) results for primary and secondary outcomes.

Variable	IF (Placebo) *n* = 11		IF (Probiotic) *n* = 15		*p*-Value ^1^	
	Baseline	12 weeks	Baseline	12 weeks	Time × group interaction	Time effect
HbA1c (mmol/mol)	42.9 ± 2.6	40.9 ± 2.9	43.1 ± 2.9	41.1 ± 1.9	0.938	<0.001
Weight (kg)	91.3 ± 13.9	86.7 ± 13.7	98.6 ± 18.4	93.7 ± 18.1	0.859	<0.001
BMI (kg/m^2^)	33.6 ± 3.7	31.9 ± 4.0	34.7 ± 4.9	33.0 ± 5.2	0.967	<0.001
Waist circumference (cm)	111.1 ± 11.0	107.5 ± 10.9	112.7 ± 16.7	106.5 ± 16.1	0.211	<0.001
Hip circumference (cm)	116.3 ± 9.8	115.5 ± 14.8	122.0 ± 13.1	117.9 ± 15.3	0.258	0.105
WHR	0.96 ± 0.10	0.94 ± 0.09	0.92 ± 0.09	0.90 ± 0.09	0.934	0.025
Neck circumference (cm)	39.1 ± 4.2	38.1 ± 3.4	40.9 ± 3.8	39.8 ± 3.8	0.971	0.048
Total body fat (kg) (DXA)	41.3 ± 9.1	37.9 ± 9.3	43.9 ± 12.3	40.3 ± 13.6	0.852	<0.001
Abdominal fat (kg) (DXA)	4.2 ± 1.1	3.7 ± 1.1	4.5 ± 1.7	4.0 ± 1.8	0.692	<0.001
Visceral fat (kg) (DXA)	2.0 ± 0.81	1.8 ± 0.7	2.1 ± 1.18	1.8 ± 1.06	0.771	<0.001
Fat-free mass (kg) (DXA)	50.8 ± 9.3	49.8 ± 9.6	55.1 ± 12.4	54.1 ± 12.4	0.992	0.005
REE (kcal/day)	1561.3 ± 162.9	1495.9 ± 193.8	1760.2 ± 422.3	1640.5 ± 317.0	0.393	0.007
RQ	0.80 ± 0.04	0.77 ± 0.06	0.79 ± 0.05	0.78 ± 0.05	0.368	0.057
Fasting glucose (mmol/L)	5.99 ± 0.35	5.76 ± 0.43	6.19 ± 0.55	6.00 ± 0.82	0.862	0.087
Fasting insulin (mU/L)	17.4 ± 8.0	13.6 ± 7.4	17.0 ± 10.8	16.8 ± 10.5	0.309	0.247
Fasting C-peptide (ng/mL)	3.74 ± 0.61	3.36 ± 0.86	3.61 ± 1.28	3.65 ± 1.61	0.149	0.234
AUC_0-120 min_ glucose (mmol/L × h)	20.9 ± 3.6	18.5 ± 3.7	19.8± 3.8	19.6 ± 3.7	0.056	0.020
AUC_0-120 min_ insulin (mU/L × h)	255 ± 81	194 ± 71	258 ± 223	248 ± 207	0.225	0.097
AUC_0-120 min_ C-peptide (pmol/L × h)	23.2 ± 3.6	22.2 ± 4.4	22.6 ± 10.5	21.6 ± 10.8	0.984	0.210
Cholesterol (mmol/L)	5.5 ± 1.1	5.1 ± 1.0	5.4 ± 1.1	5.4 ± 1.4	0.374	0.222
Triglycerides (mmol/L)	1.6 ± 0.4	1.5 ± 0.5	1.6 ± 0.7	1.4 ± 0.5	0.984	0.167
HDL (mmol/L)	1.2 ± 0.2	1.2 ± 0.2	1.3 ± 0.3	1.3 ± 0.3	0.332	0.312
LDL (mmol/L)	3.7 ± 1.1	3.5 ± 0.9	3.6 ± 1.0	3.7 ± 1.3	0.218	0.394
AST (U/L)	30.4 ± 6.4	29.4 ± 9.4	27.7 ± 11.4	26.3 ± 7.7	0.953	0.632
ALT (U/L)	35.7 ± 17.9	24.7 ± 6.0	27.3 ± 12.6	30.0 ± 12.4	0.073	0.258
Leptin (μg/mL)	16.2 ± 11.9	12.5 ± 10.7	18.9 ± 10.8	15.8 ± 9.8	0.844	0.045
TNF-α (ng/mL)	3.6 ± 1.0	4.3 ± 1.2	3.0 ± 1.2	3.4 ± 1.7	0.607	0.062
IL-6 (ng/mL)	2.4 ± 0.92	2.9 ± 0.94	3.3 ± 1.4	3.2 ± 1.1	0.131	0.264

Data presented as mean ± standard deviation. Abbreviations: OGTT, oral glucose tolerance test; IF, intermittent fasting; HbA1c, glycated hemoglobin; BMI, body mass index; WHR, waist–hip ratio; DXA, dual-energy X-ray absorptiometry; BMD, bone mineral density; REE, resting energy expenditure; RQ, respiratory quotient; AUC, area under the curve; HDL, high-density lipoprotein; LDL, low-density lipoprotein; AST, aspartate aminotransferase; ALT, alanine aminotransferase; TNF-α, tumor necrosis factor; IL-6, interleukin 6. ^1^ Repeated measures analysis of variance.

**Table 3:** Comparison of energy and macronutrient intake at baseline and after 12 weeks of IF in the IF placebo and probiotic groups.

Measurement	IF (Placebo) *n* = 8		IF (Probiotic) *n* = 11		*p* Value ^1^	
	Baseline	12 Weeks	Baseline	12 Weeks	Time x Group Interaction	Time Effect
Energy (kcal/d) including fasting days	2222 ± 394	1693 ± 316	2374 ± 731	1778 ± 624	0.832	0.002
Energy (kcal/kg/d) including fasting days	25.0 ± 4.7	20.5 ± 4.4	24.9 ± 8.6	19.1 ± 4.4	0.713	0.008
Energy (kcal/d) excluding fasting days	2222 ± 394	1863 ± 383	2374 ± 731	2061 ± 866	0.891	0.058
Energy (kcal/d) on fasting days	N/A	755.4 ± 207.8	N/A	651.6 ± 147.7	0.206	N/A
Protein (g/d)	92.9 ± 24.1	77.0 ± 14.6	108.7 ± 43.1	87.8 ± 34.1	0.767	0.039
Protein (g/kg/d)	1.1 ± 0.3	0.9 ± 0.2	1.1 ± 0.4	0.9 ± 0.2	0.672	0.079
Protein (% TE)	16.8 ± 2.2	18.9 ± 4.8	18.7 ± 4.1	20.4 ± 5.3	0.885	0.193
Carbohydrate (g/d)	219.5 ± 17.1	169.6 ± 62.8	226.1 ± 86.8	160.4 ± 81.6	0.696	0.010
Carbohydrate (% TE)	40.5 ± 9.6	38.0 ± 11	37.5 ± 5.7	35.8 ± 10.7	0.870	0.349
Total fat (g/d)	98.9 ± 34.6	70.7 ± 13.3	101.4 ± 35.5	77.5 ± 32.6	0.799	0.005
Total fat (% TE)	38.1 ± 9.4	37.5 ± 7.8	37.6 ± 4.8	38.2 ± 6.9	0.701	0.990
Saturated fat (% TE)	16.1 ± 4.3	14.1 ± 2.5	16.3 ± 2.4	14.5 ± 2.9	0.884	0.039
Saturated fat (% total fat)	47.5 ± 6.0	42.1 ± 6.3	49.4 ± 6.7	43.1 ± 4.9	0.766	0.002
Monounsaturated fat (% total fat)	37.0 ± 4.5	41.4 ± 5.0	36.1 ± 3.7	38.6 ± 4.5	0.474	0.016
Polyunsaturated fat (% total fat)	15.5 ± 7.1	16.5 ± 4.0	14.5 ± 5.4	18.3 ± 6.3	0.301	0.094
Total sugars (g/d)	101 ± 33.7	84.6 ± 37.1	86.2 ± 35.4	72.1 ± 31.6	0.919	0.172
Fiber (g/d)	27.5 ± 4.2	22.2 ± 7.8	26.5 ± 11.4	23.4 ± 6.3	0.645	0.095
Alcohol (g/d)	3.8 ± 9.4	3.6 ± 8.2	9.5 ± 12.1	5.8 ± 10.9	0.203	0.172

Data presented as mean ± standard deviation. Abbreviations: IF, intermittent fasting; g/d, grams per day; % TE, percentage of total energy. ^1^ Repeated measures analysis of variance. All nutrient measures include fasting days unless otherwise specified.

**Table 4:** Results of depression scores measured by the Patient Health Questionnaire 9 (PHQ9) questionnaire, anxiety scores measured by the General Anxiety Disorder (GAD) questionnaire and quality of life scores measured by the Short Form 12 (SF-12) questionnaire at baseline and 12 weeks in the IF placebo and IF probiotic groups.

	IF (Placebo) *n* = 10		IF (Probiotic) *n* = 15		*p*-Value ^1^	
	Baseline	12 Weeks	Baseline	12 Weeks	Time x Group Interaction	Time Effect
PHQ9 ^2^	4.5 (0–12)	1.5 (0–10)	3 (0–15)	4 (0–6)	0.845	0.003
GAD ^2^	3.5 (0–16)	3.5 (0–17)	2 (0–12)	1 (0–10)	0.137	0.132
SF-12 domains:						
Physical Functioning	67.5 ± 26.5	77.5 ± 24.9	78.3 ± 22.9	75.0 ± 28.4	0.233	0.546
Role Physical	73.8 ± 17.1	85.0 ± 21.1	82.5 ± 22.6	80.8 ± 17.6	0.179	0.314
Bodily Pain	82.5 ± 26.5	80.0 ± 30.7	68.3 ± 29.1	80.0 ± 21.6	0.201	0.413
General Health	63.0 ± 23.2	75.5 ± 21.5	59.0 ± 28.0	69.7 ± 19.9	0.834	0.013
Vitality	60.0 ± 17.5	57.5 ± 23.7	51.7 ± 25.8	60.0 ± 18.4	0.269	0.548
Social Functioning	85.0 ± 21.1	85.0 ± 17.5	76.7 ± 22.1	91.7 ± 20.4	0.050	0.050
Role Emotional	81.3 ± 14.7	80.0 ± 16.9	83.0 ± 20.6	88.4 ± 21.6	0.256	0.476
Mental Health	73.8 ± 13.8	72.5 ± 12.9	69.2 ± 14.8	80.0 ± 10.4	0.007	0.028

Values expressed as mean ± standard deviation unless otherwise specified. Abbreviations: IF, intermittent fasting. ^1^ Repeated measures analysis of variance. ^2^ Values expressed as median (range).
